# Rhinovirus C and Respiratory Exacerbations in Children with Cystic Fibrosis

**DOI:** 10.3201/eid1606.100063

**Published:** 2010-06

**Authors:** Marina B. de Almeida, Rodrigo M. Zerbinati, Adriana F. Tateno, Cristina M. Oliveira, Renata M. Romão, Joaquim C. Rodrigues, Cláudio S. Pannuti, Luiz Vicente F. da Silva Filho

**Affiliations:** Hospital das Clínicas da Faculdade de Medicina de Universidade de São Paulo, São Paulo, Brazil (M.B. de Almeida, J.C. Rodrigues, L.V.F. da Silva Filho); Universidade de São Paulo, São Paulo (R.M. Zerbinati, A.F. Tateno, C.M. Oliveira, R.M. Romão, C.S. Pannuti, L.V.F. da Silva Filho)

**Keywords:** Rhinovirus, viruses, cystic fibrosis, respiratory infections, children, Brazil, dispatch

## Abstract

To investigate a possible role for human rhinovirus C in respiratory exacerbations of children with cystic fibrosis, we conducted microbiologic testing on respiratory specimens from 103 such patients in São Paulo, Brazil, during 2006–2007. A significant association was found between the presence of human rhinovirus C and respiratory exacerbations.

Cystic fibrosis (CF) is an autosomal inherited disease characterized by recurrent and chronic respiratory infections that ultimately lead to the need for a lung transplant early in life or to death (*1*). The role of bacterial infections in CF is well established, and most treatments focus on eradication or suppression of bacterial infections (mainly those caused by *Pseudomonas aeruginosa*) (*1*).

Respiratory viruses such as respiratory syncytial virus (RSV) and influenza also seem to cause early damage or increase the risk for respiratory exacerbations (*2**,**3*) in these patients. However, the role of newly described respiratory viruses is not well known. Infection of these patients with human rhinovirus (HRV), a member of the family *Picornaviridae*, has been described. Although some studies have suggested a substantial pathogenic role for these viruses (*3*), controversy still exists (*4*).

Recently a new clade of human rhinovirus, named rhinovirus C (*5*), was identified through molecular methods. This new clade has been found throughout the world (*6*), and some studies have attributed severe respiratory infections in children to this agent (*7*,*8*). We investigated whether this agent played a role in the respiratory exacerbations of children and adolescents with CF who attended the Instituto da Crinaça, Hospital das Clínicas da Faculdade de Medicina da Universidade de São Paulo.

## The Study

A total of 103 CF patients (49 girls, 54 boys; median age 8.9 years; age range 3.8 months–17.8 years) were enrolled in the study from September 6, 2006 through September 4, 2007. Nasopharyngeal aspirates or nasal mucus specimens for viral investigation, as well as sputum or oropharyngeal samples for microbiology culture, were collected during scheduled visits or unscheduled visits on 408 occasions, with a median ± SD of 4 ± 1.74 visits per patient (range 1–9 visits).

Clinical and lung function data were obtained at all visits. Exacerbation of respiratory disease was defined as the presence of >2 of the following signs or symptoms: fever, increase in the amount of secretion or cough intensity, change in sputum’s color, worsening of dyspnea, loss of appetite, a decrease of forced expiratory volume in 1 s >10%, and weight loss.

Total nucleic acids were extracted from nasopharyngeal samples by using a QIAmp Viral RNA Mini Kit (QIAGEN, Hamburg, Germany), according to manufacturer’s instructions. Reverse transcription was conducted with High Capacity cDNA Archive Kit (Applied Biosystems, Foster City, CA, USA) by using 20 μL of the previously extracted RNA. Respiratory viruses were identified by individual reverse transcription–PCRs or PCRs selective for RSV; influenza viruses A and B; human parainfluenza viruses 1, 2, and 3; human coronavirus; human metapneumovirus; adenovirus; human bocavirus; picornavirus; and the β-actin gene (*9*). For picornavirus, we used the primer pair OL26–OL27, which include a portion of the 5′ noncoding region (NCR) common to all picornaviruses, in the same conditions previously described (*10*). After sequence amplification, products were examined by capillary electrophoresis in an automated DNA sequencer (MegaBace, General Electric Healthcare–Amersham Biosciences, Little Chalfont, UK), and results were visualized through the MegaBace FragmentProfiler software, which discriminates fragment sizes and fluorescent intensities.

Samples in which picornavirus cDNA had been identified were submitted to a TaqMan-based real-time PCR protocol (*11*) to identify HRV and enterovirus. Only samples in which rhinovirus had been identified by real-time PCR were submitted to 5′ ΝCR sequencing with an ABI 377 automated sequencer (Applied Biosystems), and results were submitted to the GenBank database, accession nos. GU933027–GU933118. Sequencing of the 5′ NCR region has been shown to accurately discriminate among subtypes of rhinovirus, including genotypes C and A2 (*12*). All sequencing chromatograms obtained were edited manually to obtain contiguous fragments (contigs), by using Sequence Navigator software (Applied Biosystems). All sequences were screened at the National Center for Biotechnology Information website by using the Basic Local Alignment Search Tool (BLAST) (http://blast.ncbi.nlm.nih.gov/Blast.cgi). HRV genotype was confirmed by phylogenetic analysis as described below. Sequences generated and a set of reference strains representative of HRV genotypes, available in the GenBank database, were aligned by using ClustalW (www.ebi.ac.uk/Tools/clustalw2). Minor manual adjustments were made to improve the alignment with BioEdit software (www.mbio.ncsu.edu/BioEdit/BioEdit.html). The sequence of echovirus 11 was used as the outgroup. Phylogenetic analysis was performed with PAUP* version 4b10 (http://paup.csit.fsu.edu). Neighbor-joining and maximum-likelihood trees were constructed on the basis of appropriate nucleotide substitution models determined by Modeltest v3.7 (University of Vigo, Vigo, Spain). Bootstrapping was assessed by using 1,000 replicates. Trees were visualized by using the TreeView program (www.taxonomy.zoology.gla.ac.uk/rod/treeview.html).

To account for correlations among samples from the same patient, we used binomial generalized linear models to identify the virologic variables associated with the main endpoints (respiratory exacerbation and hospital admission). Results were presented as odds ratios (ORs) and 95% confidence intervals.

At least 1 respiratory virus was identified in 203 (49.8%) of 408 samples; rhinovirus was the main identified agent (139 samples, 34.1%). The results of virus identification, in relation to clinical status, are shown in Table 1. Co-infections were found in only 26 samples (6.4%); rhinovirus was the most frequent agent.

**Table 1:** Respiratory viruses and clinical status of 103 children with cystic fibrosis , Brazil, 2006–2007

Virus	No. samples collected during routine visits, n = 266	No. samples collected during respiratory exacerbations, n = 142	Total no. (%) samples
Rhinovirus	91	48	139 (34.1)
Enterovirus	13	11	24 (5.9)
Human bocavirus	14	9	23 (5.6)
Human coronavirus	13	6	19 (4.7)
Respiratory syncytial virus	7	8	15 (3.7)
Influenza A	1	3	4 (1.0)
Human metapneumovirus	1	2	3 (0.7)
Influenza B	1	0	1 (0.2)
Parainfluenza 1	0	1	1 (0.2)
Parainfluenza 2	0	1	1 (0.2)
Parainfluenza 3	0	1	1 (0.2)
Adenovirus	0	1	1 (0.2)

**Figure 1:**
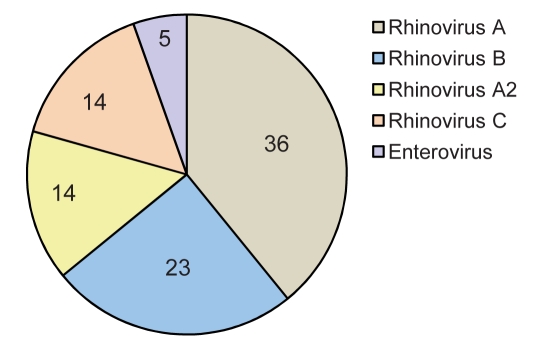
Results of 5′ noncoding region sequencing of 93 samples with identification of human rhinovirus by real-time reverse transcription–PCR, obtained from samples from 103 children with cystic fibrosis, Brazil, 2006–2007.

**Figure 2:**
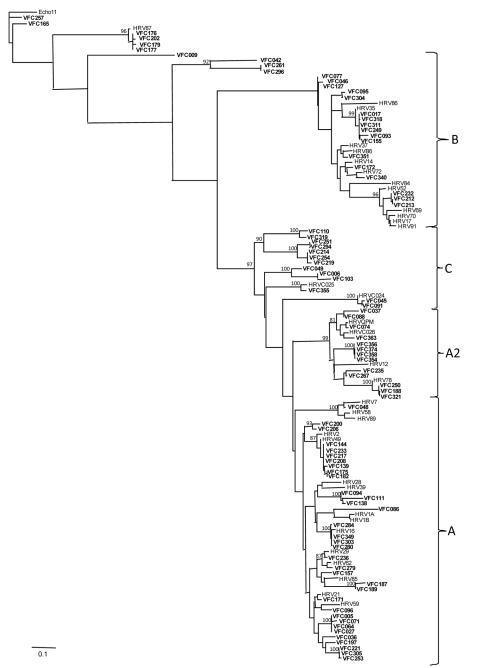
Representative maximum-likelihood phylogenetic tree of a partial 5′ noncoding region of human rhinovirus generated with general time reversible substitution model, including gamma distribution shape parameter. Reference human rhinovirus (HRV) genotypes were obtained from the GenBank database. Echovirus 11 was defined as the outgroup. Virus isolates obtained in this study are indicated by **boldface** and are labeled VFC. Bootstrap values >70% in the key branches are depicted. Scale bar indicates nucleotide substitutions per site.

Sequencing was performed in 93 of 139 samples that were positive for rhinovirus and showed a predominance of genotype A (36 samples; 38.7%) (Figure 1). Rhinovirus subtypes A2 and C were identified in 14 samples each. Isolates in 3 samples were identified as HRV87, coxsackievirus, and echovirus. Therefore, 5 isolates were enteroviruses, which indicates that the TaqMan–based real-time PCR platform misidentified them. Sequencing was not successful in 1 sample. A maximum-likelihood phylogenetic tree of our samples and reference HRV sequences was constructed (Figure 2).

Patients were examined during acute exacerbation of respiratory disease on 142 occasions, and patients required hospital admission on 31 occasions. The identification of respiratory viruses was not associated with pulmonary exacerbations. Because rhinovirus was the main agent identified among stable patients, we verified the effects of respiratory viruses, excluding rhinovirus from the analysis. A significant association with respiratory exacerbation was found (OR 1.195, p = 0.010) (Table 2).

**Table 2:** Virologic test results and respiratory exacerbation outcome for 103 children with cystic fibrosis, Brazil, 2006–2007*

Virus	OR (95% CI)	p value
Any respiratory virus	1.063 (0.979–1.154)	0.144
Rhinovirus	1.020 (0.931–1.117)	0.666
Any respiratory virus except rhinoviruses	1.195 (1.043–1.369)	0.010
Rhinovirus A2 or C (excluding samples not sequenced)	1.213 (1.024–1.436)	0.025

*Estimates from binomial, generalized linear models. OR, odds ratio; CI, confidence interval.

In contrast, when looking at rhinovirus subtypes, we noticed that identification of rhinovirus subtypes A2 or C was also significantly associated with respiratory exacerbations (OR 1.213) (Table 2). Identification of influenza A was the only variable associated with an increase in the risk for hospital admission (OR 1.988, 95% confidence interval 1.238–3.194, p = 0.004).

## Conclusions

These new HRV genotypes were initially described in samples from patients with influenza-like illnesses (*13*). Recently, evidence has been increasing for the involvement of HRV-C in severe respiratory conditions such as bronchiolitis in infants (*7*) and in exacerbation of asthma (*14*). However, these studies that attempted to clarify the pathogenicity of these new species did not include control groups or nonsymptomatic persons.

In the study reported here, we obtained samples from patients during routine visits and exacerbations, which enabled us to identify a distinct role of different HRV subtypes. Our findings, however, cannot be extrapolated to infants with CF, which were underrepresented in our study. These infants may be at greater risk of HRV-C respiratory infections as they are for with RSV infections (*2*).

Previous studies of respiratory virus infections in CF patients provided conflicting results on the potential effect of rhinovirus. Smyth et al (*15*), who evaluated respiratory exacerbations in CF patients, identified rhinovirus as the leading agent, with exacerbations following a more benign course but resulting in greater use of intravenous antimicrobial drugs (*15*). Olesen et al. (*4*) studied 75 CF patients for 1 year and used PCR to investigate the presence of 7 different viruses in sputum or laryngeal aspirates. The group reported that viral infections did not reduce lung function or increase respiratory symptoms. Rhinovirus was by far the leading agent identified throughout their study (*4*). A more recent study by Wat et al. (*3*), in which they used real-time nucleic acid sequence–based amplification in conjunction with molecular markers to investigate the presence of 9 respiratory viruses in children with CF, described an association of viral infections with respiratory exacerbations, particularly those caused by influenza A, influenza B, and rhinovirus (*3*).

We report infections by the novel rhinovirus subtypes A2 and C in CF patients. A significant association was found between the presence of these agents and respiratory exacerbations. Our findings indicate the need for further investigation of HRV-C in CF patients.

## References

[1] Ratjen F, Doring G. Cystic fibrosis. Lancet. 2003;361:681–9. 10.1016/S0140-6736(03)12567-6 12606185

[2] Armstrong D, Grimwood K, Carlin JB, Carzino R, Hull J, Olinsky A, Severe viral respiratory infections in infants with cystic fibrosis. Pediatr Pulmonol. 1998;26:371–9. 10.1002/(SICI)1099-0496(199812)26:6<371::AID-PPUL1>3.0.CO;2-N 9888211

[3] Wat D, Gelder C, Hibbitts S, Cafferty F, Bowler I, Pierrepoint M, The role of respiratory viruses in cystic fibrosis. J Cyst Fibros. 2008;7:320–8. Epub 2008 Feb 6. 10.1016/j.jcf.2007.12.002 18255355 PMC7105190

[4] Olesen HV, Nielsen LP, Schiotz PO. Viral and atypical bacterial infections in the outpatient pediatric cystic fibrosis clinic. Pediatr Pulmonol. 2006;41:1197–204. 10.1002/ppul.20517 17058280

[5] McErlean P, Shackelton LA, Andrews E, Webster DR, Lambert SB, Nissen MD, Distinguishing molecular features and clinical characteristics of a putative new rhinovirus species, human rhinovirus C (HRV C). PLoS One. 2008; 3:e1847. 10.1371/journal.pone.0001847 PMC2268738 18382652

[6] Briese T, Renwick N, Venter M, Jarman RG, Ghosh D, Kondgen S, Global distribution of novel rhinovirus genotype. Emerg Infect Dis. 2008;14:944–7. 10.3201/eid1406.080271 18507910 PMC2600308

[7] Lau SK, Yip CC, Tsoi HW, Lee RA, So LY, Lau YL, Clinical features and complete genome characterization of a distinct human rhinovirus (HRV) genetic cluster, probably representing a previously undetected HRV species, HRV-C, associated with acute respiratory illness in children. J Clin Microbiol. 2007;45:3655–64. 10.1128/JCM.01254-07 17804649 PMC2168475

[8] Wisdom A, Leitch EC, Gaunt E, Harvala H, Simmonds P. Screening respiratory samples for detection of human rhinoviruses (HRVs) and enteroviruses: comprehensive VP4–VP2 typing reveals high incidence and genetic diversity of HRV species C. J Clin Microbiol. 2009;47:3958–67. 10.1128/JCM.00993-09 19828751 PMC2786677

[9] da Silva Filho LVF, de Almeida MB, Flores TR, Tomikawa S, Wickbold D, Tateno AF, Clinical and functional impact of acute viral respiratory infections in cystic fibrosis. Pediatr Pulmonol. 2008;43:344.

[10] Papadopoulos NG, Sanderson G, Hunter J, Johnston SL. Rhinoviruses replicate effectively at lower airway temperatures. J Med Virol. 1999;58:100–4. 10.1002/(SICI)1096-9071(199905)58:1<100::AID-JMV16>3.0.CO;2-D 10223554

[11] Deffernez C, Wunderli W, Thomas Y, Yerly S, Perrin L, Kaiser L. Amplicon sequencing and improved detection of human rhinovirus in respiratory samples. J Clin Microbiol. 2004;42:3212–8. 10.1128/JCM.42.7.3212-3218.2004 15243084 PMC446277

[12] Kiang D, Kalra I, Yagi S, Louie JK, Boushey H, Boothby J, Assay for 5′ noncoding region analysis of all human rhinovirus prototype strains. J Clin Microbiol. 2008;46:3736–45. 10.1128/JCM.00674-08 18753359 PMC2576622

[13] Lamson D, Renwick N, Kapoor V, Liu Z, Palacios G, Ju J, MassTag polymerase-chain-reaction detection of respiratory pathogens, including a new rhinovirus genotype, that caused influenza-like illness in New York State during 2004–2005. J Infect Dis. 2006;194:1398–402. 10.1086/508551 17054069 PMC7110122

[14] Khetsuriani N, Lu X, Teague WG, Kazerouni N, Anderson LJ, Erdman DD. Novel human rhinoviruses and exacerbation of asthma in children. Emerg Infect Dis. 2008;14:1793–6. 10.3201/eid1411.080386 18976575 PMC2630738

[15] Smyth AR, Smyth RL, Tong CY, Hart CA, Heaf DP. Effect of respiratory virus infections including rhinovirus on clinical status in cystic fibrosis. Arch Dis Child. 1995;73:117–20. 10.1136/adc.73.2.117 7574853 PMC1511210

